# Satellite data based estimation of methane emissions from rice paddies in the Sanjiang Plain in northeast China

**DOI:** 10.1371/journal.pone.0176765

**Published:** 2017-06-06

**Authors:** Minmin Sun, Yuan Zhang, Jing Ma, Wenping Yuan, Xianglan Li, Xiao Cheng

**Affiliations:** 1 State Key Laboratory of Earth Surface Processes and Resource Ecology, Beijing Normal University, Beijing, China; 2 College of Global Change and Earth System Science, Beijing Normal University, Beijing, China; 3 Joint Center for Global Change Studies, Beijing Normal University, Beijing, China; 4 Key Laboratory of Geographical Information Science (Ministry of Education), East China Normal University, Shanghai, China; 5 School of Geographic Sciences, East China Normal University, Shanghai, China; 6 State Key Laboratory of Soil and Sustainable Agriculture, Institute of Soil Science, Chinese Academy of Sciences, Nanjing, Jiangsu, China; 7 School of Atmospheric Sciences, Sun Yat-sen University, Zhuhai, Guangdong, China; Montana State University Bozeman, UNITED STATES

## Abstract

The Sanjiang Plain (SJP), one of the major rice producing regions in China, is an important source of methane (CH_4_) emissions. However, there have been large uncertainties in the estimates of CH_4_ emissions from this area during the past few years. In this study, we estimated CH_4_ emissions using a process-based model derived by rice area, CH_4_ flux, land surface temperature (LST), and the ratio of precipitation (P) and evapotranspiration (ET) in rice paddies in the SJP during the rice growing seasons in 2000, 2006, and 2010, respectively. The results showed that the total area of rice fields was 1.64 million ha in 2010, which was approximately 35 and 13% higher than in 2000 and 2006, respectively. The average LST was 22.1°C in 2000 which was higher than in 2006 (21.6°C) and 2010 (21.5°C). Monthly ET and P displayed similar seasonal and annual variability. The monthly ET was 61.7, 66.5, and 63.0 mm month^-1^ and P was 85.1, 80.6 and 85.9 mm month^-1^ during the rice growing seasons in 2000, 2006, and 2010, respectively. The averaged CH_4_ flux rates were 24.83, 24.63, and 24.59 ton km^-2^, and the estimated mean annual CH_4_ emissions from rice paddies were 0.30, 0.36, and 0. 40 Tg yr^-1^ in 2000, 2006, and 2010, respectively. The CH_4_ emissions displayed obvious spatial variations that decreased from east to west in the SJP, and were mainly affected by temperature. The results will improve our understanding of the inter-annual and spatial variations of CH_4_ emissions and provide a more accurate regional budget of CH_4_ emissions from rice paddies in the Sanjiang Plain.

## Introduction

The rising atmospheric concentrations of methane (CH_4_) play an important role in global warming. Methane has a relative global warming potential 35 times higher than that of carbon dioxide (CO_2_), with unique radiative properties and a short residence time in the atmosphere [1, 2]. Agriculture is an important source of CH_4_, and accounts for approximately 52% of global anthropogenic CH_4_ emissions [3]. Rice paddies has been identified as one of major agricultural source of atmospheric CH_4_ [4, 5]. Global CH_4_ emissions from rice fields are 21–30 Tg yr^-1^ [6], and account for more than 10% of the total CH_4_ emissions in the atmosphere [7]. Therefore, it is important to estimate CH_4_ emissions, and to reduce the uncertainties in CH_4_ emissions from rice paddies at regional and global scales.

The Sanjiang Plain (SJP) which is drained by the Songhuajiang, Ussuri, and Heilongjiang rivers, is a typical paddy rice region in China [8–11]. Previous reports have indicated that the area of paddy rice fields increased from 1.07 million ha in 2000 to 1.43 million ha in 2006 [11, 12], which represents a dramatic increase of approximately 35%. By 2010, the area of paddy rice fields in the SJP region accounted for more than 10% of the total rice cropping area in China [12, 13]. Many efforts have been made to characterize CH_4_ emissions from rice paddy in the SJP [12–15]. Early field measurements indicated that mean flux rates of CH_4_ emissions from rice paddy in the SJP varied from 0.05 to 24.37 mg m^-2^ h^-1^, with a mean value of 6.67 mg m^-2^ h^-1^ [15]. Wang et al. [13] estimated CH_4_ emissions using the area expansion method and obtained an estimation of 0.10 Tg yr^-1^ for cold paddy fields in the SJP regions. However, area expansion method may ignore the importance of environmental and soil conditions in the processes of CH_4_ generation from rice fields [16–18]. With an understanding of the processes of CH_4_ generation, the Denitrification and Decomposition (DNDC) model was developed and further modified to simulate CH_4_ emissions from rice fields in the SJP [11, 12, 19, 20]. Zhang et al. [11, 12] used the DNDC model to estimate CH_4_ emissions as 0.50, 0.71, and 0.49 Tg yr^-1^ from paddy fields in the SJP in 2000, 2006, and 2010 with a cell size of 10 × 10 km. Attempts have been made to explain the regional variations of CH_4_ emissions in these studies, but the input data of these models has a low spatial and temporal resolution.

Remote sensing technology is capable of providing more accurate paddy field data, with spatially explicit information when compared to the inadequate and problematic census data [21, 22]. The rice paddy cover was mapped based on nine Landsat thematic mapper (TM) images acquired in 2006, with a total area of 1.44 million ha using the visual interpretation method [12]. Remote sensing techniques provide near-real-time and high spatial resolution data products related to global environmental variables, such as land surface temperature (LST) and evapotranspiration (ET). Agarwal et al. [23] presented a large—scale model integrating field data with Moderate Resolution Imaging Spectroradiometer (MODIS) derived LST and ET data to estimate CH_4_ emissions from rice paddies, which provided better insights into the regional variations of CH_4_ emissions. Akumu et al. [24] improved this model to estimate CH_4_ emissions from wetlands in North-Eastern New South Wales. However, there have been few estimates of CH_4_ emissions from rice paddies in China based on remote sensing data and field observations, from which CH_4_ emissions could be extrapolated from the site scale to a larger spatial dimension.

Driven by the market demand for high quality rice in China, more dryland crop such as corn or soybeans have been converted to wetland crops (mainly rice) in northern China in recent decades. This kind of rapid land-use transformation could alter the national greenhouse gases (GHGs) inventory currently and even in the near future. Updating the land-use and mapping the consequent GHGs emissions are becoming an urgent task for quantifying the global warming impacts induced by cropland. In this present study, we aim to: (1) analyze the spatial characteristics of CH_4_ emissions and estimate the CH_4_ emissions from SJP based on satellite data; and (2) explore the impact of various factors on CH_4_ emissions during the rice growing season at regional scale.

## Materials and methods

### Study area

**Fig 1:**
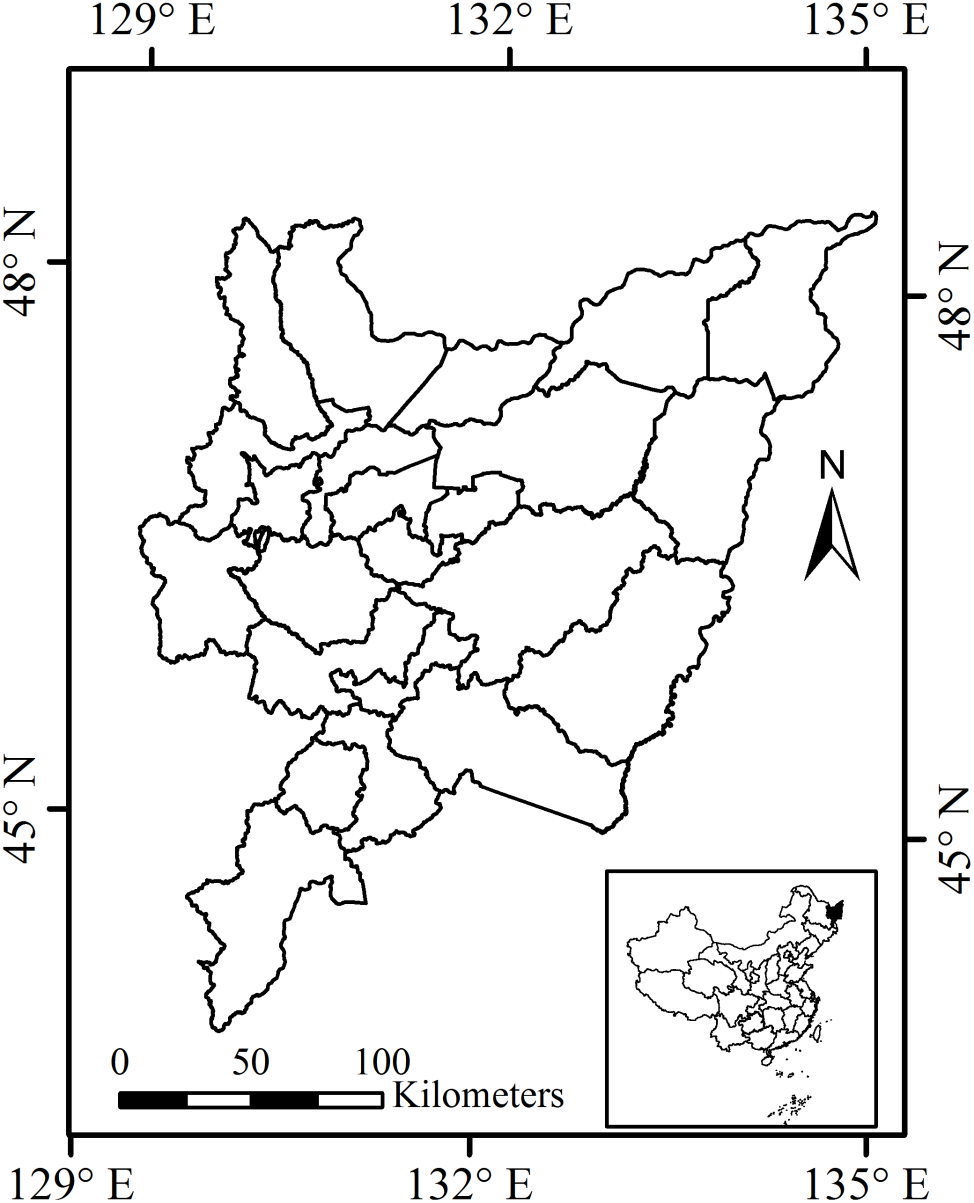
Location of the Sanjiang Plain (SJP) in China.

This study was conducted in the SJP, which is the highest latitude region for rice cultivation in the world. The SJP covers an area of 10.93 million ha and is located in the eastern part of Heilongjiang Province. It is located between 48.5° and 43.8°N latitude and 129.2° and 135.1°E longitude (Fig 1). The region is a low plain that features an alluvial formation due to the confluence of the Heilongjiang, Songhua, and Ussuri rivers, and lies at 45 to 60 m geographic elevation above sea level with a gentle and flat topographic relief. During the period of 1980–2010, the annual mean temperature in the area ranged from 2.6 to 5.2°C, and annual precipitation (P) ranged from 330 to 850 mm and was mainly concentrated in June through August [11].

The SJP has a temperate humid and semi humid continental monsoon climate. Temperatures and P are high in the summer in this region. Crops can obtain sufficient calories and water in the vigorous growth period. In the 1990s, the large-scale development of irrigated rice cultivation began in the region. According to remote sensing image data, the region's rice planting area reached 1.64 million ha in 2010. The single-season rice is grown predominantly from May to October in this region.

### Data source

To obtain the observed mean CH_4_ flux for paddy rice in the SJP during the growing season, we screened the Chinese National Knowledge Infrastructure and Web of Science for relevant papers. In the studies identified, the observed CH_4_ fluxes were measured using a static chamber-gas chromatograph technique at the Sanjiang Mire-Wetland Experimental Station of the Chinese Ecosystem Research Network (133°31′ E, 47°35′ N) from 2001–2006. Each sampling point was located at a distance of 3 m from the previous sampling point. Gas samples were measured twice at one week intervals in the entire paddy rice growing season from late May to early October.

Temperature and ET data were obtained from MODIS (S1 File). The MOD11 daily data was retrieved at 1 km pixels by the generalized split-window algorithm in seven thermal infrared bands. The MOD16 ET products can be used to calculate the regional soil water status; hence, it provides key information for water resource management. With long-term ET data, the effects of climate changes on regional water resources and land surface energy changes can be quantified. According to the geographical position of the SJP, we choose the satellite track numbers, h26v04 and h27v04, which fully covered the whole area. We made use of the improved 8-day MOD11A2 (LST) and monthly MOD16A2 (ET) products from the National Aeronautics and Space Administration website (http://modis.gsfc.nasa.gov/). We estimated 8-day LST, and used these estimates to obtain mean monthly temperature. P data was derived from the Goddard Earth Science Data and Information Services Center measured by the tropical rainfall measuring mission (TRMM) satellite (S1 File) (http://trmm.gsfc.nasa.gov/). The rice planting area in the SJP was determined by Landsat thematic mapper (TM) remote sensing imagery provided by Yuan Zhang acquired in 2000–2010 (S1 Table). These Landsat TM images accessed from the EarthExplorer Interface (http://edcsns17.cr.usgs.gov/EarthExplorer/).

### Estimation of CH_4_ emissions

A model based on the CH_4_ production process was used to estimate the CH_4_ emission. In this study, CH_4_ emissions due to various factors were considered, including soil moisture, rice extent, and temperature. Temperature related factors are used to model the change in microbial activity and growth rate is used as a function of temperature [23]. Soil moisture content directly affects CH_4_ production by creating a low redox potential and anaerobic soil environment for methanogens [23, 25].

To determine emissions of CH_4_, we developed a simple model that was modified from Agarwal [23] and Akumu [24]:

$${\text{E}}_{\text{C}{\text{H}}_{4}}={\text{E}}_{\text{obs}}\cdot \text{Ft}\cdot \text{A}\cdot \text{fw}$$ (1)

Where E_obs_ is the observed CH_4_ flux from rice, Ft is an expression of the temperature, A is the area of rice, and fw is ratio of precipitation to evaporation.

Ft is calculated by

$$\text{Ft}=\frac{\text{F}(\text{Ts})}{\bar{\text{F}(\text{Ts})}}$$ (2)

$$\text{F}(\text{Ts})=\frac{{\text{e}}^{0.334(\text{Ts}-23)}}{1+{\text{e}}^{0.334(\text{Ts}-23)}}$$ (3)

Ts=LST⋅0.02−273.15 (4)

Where LST is land surface temperature, $\bar{\text{F}(\text{Ts})}$ is the mean of F(Ts) from rice paddies in Sanjiang Plain.

fw is calculated by:

$$\text{fw}=\left\{ \begin{array}{l} \frac{\text{P}}{\text{ET}}\;(\text{P}<\text{ET})\\ \;1(\text{P}>\text{ET}) \end{array}\right.$$ (5)

Where P is precipitation and ET is evapotranspiration. If precipitation is higher than evaporation, it is assumed that the water are saturated in soil for a given period [24].

We simulated CH_4_ emissions by modifying the model pixel by pixel across the whole rice paddy area in the SJP. We estimated CH_4_ emission rates and total CH_4_ emissions at the cell scale in a 1 × 1 km grid.

### Data analysis

In this article, all data processing is based on ArcGIS 10.1 (Esri Inc., USA). We have carried on the coordinate system definition, resampling and basic spatial processing. The spatial distribution of CH_4_ emission in the SJP is presented using the ArcGIS 10.1 (Esri Inc., USA) driven model. The statistical analyses of the CH_4_ emission were carried out by zonal statistics using ArcGIS 10.1 (Esri Inc.,USA), the seasonal variation of CH_4_ emission fluxes were drawn using SigmaPlot 12.5 (Systat Software Inc., USA).

## Results

### Observed CH_4_ flux and the area of rice paddies

**Fig 2:**
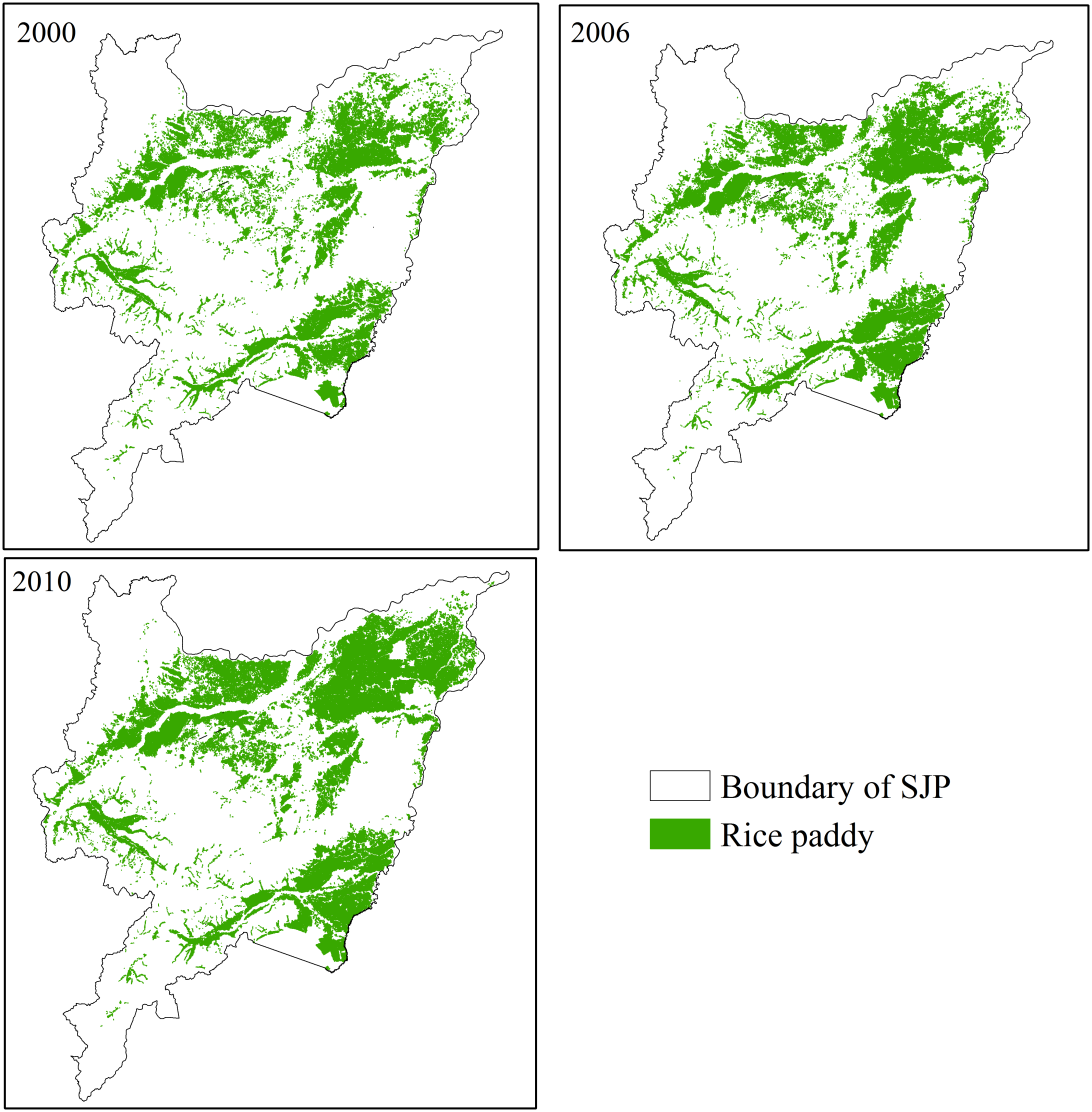
The distribution of rice paddies in the Sanjiang Plain (SJP) in 2000, 2006, and 2010.

We selected the average methane emissions from paddy fields across the entire growing season in different experimental fields in the SJP from published paper as observed CH_4_ flux [13–15, 26]. The mean CH_4_ emission rate under conventional fertilizer application in the SJP was 5.92 mg m^-2^ h^-1^. The main rice planting areas were located near river basins, including upstream of Anbang River, Songhua River, Raoli River and Xingkai Lake, and Muleng River Basin (Fig 2). These regions have large areas available for rice cultivation because the fertile soil and provision of irrigation facilities provide favorable conditions for rice planting. Using visual interpretation technique on clear TM images in 2000–2010, we map the rice paddy cover in the domain 2000, 2006, and 2010 [20, 21]. The area of rice paddy planted in the SJP was 1.21, 1.45, and 1.64 million ha in 2000, 2006, and 2010, respectively, which was almost identical to the values reported in previous studies [20, 21]. The SJP has contained more than 1.2 million ha of paddy rice since 2000.

The increase trend in the change of total area of rice paddy was observed within the SJP, with the increasement of 2.8 and 2.9% during the periods of 2000 to 2006, and from 2006 to 2010, respectively. The extension of the rice area was mainly concentrated in the northeast of Songhua River and Flexure River Basin, and gradually expanded further northeast in the 10—year from 2000 to 2010.

### Environmental factors controlling CH_4_ emissions from rice paddies

**Fig 3:**
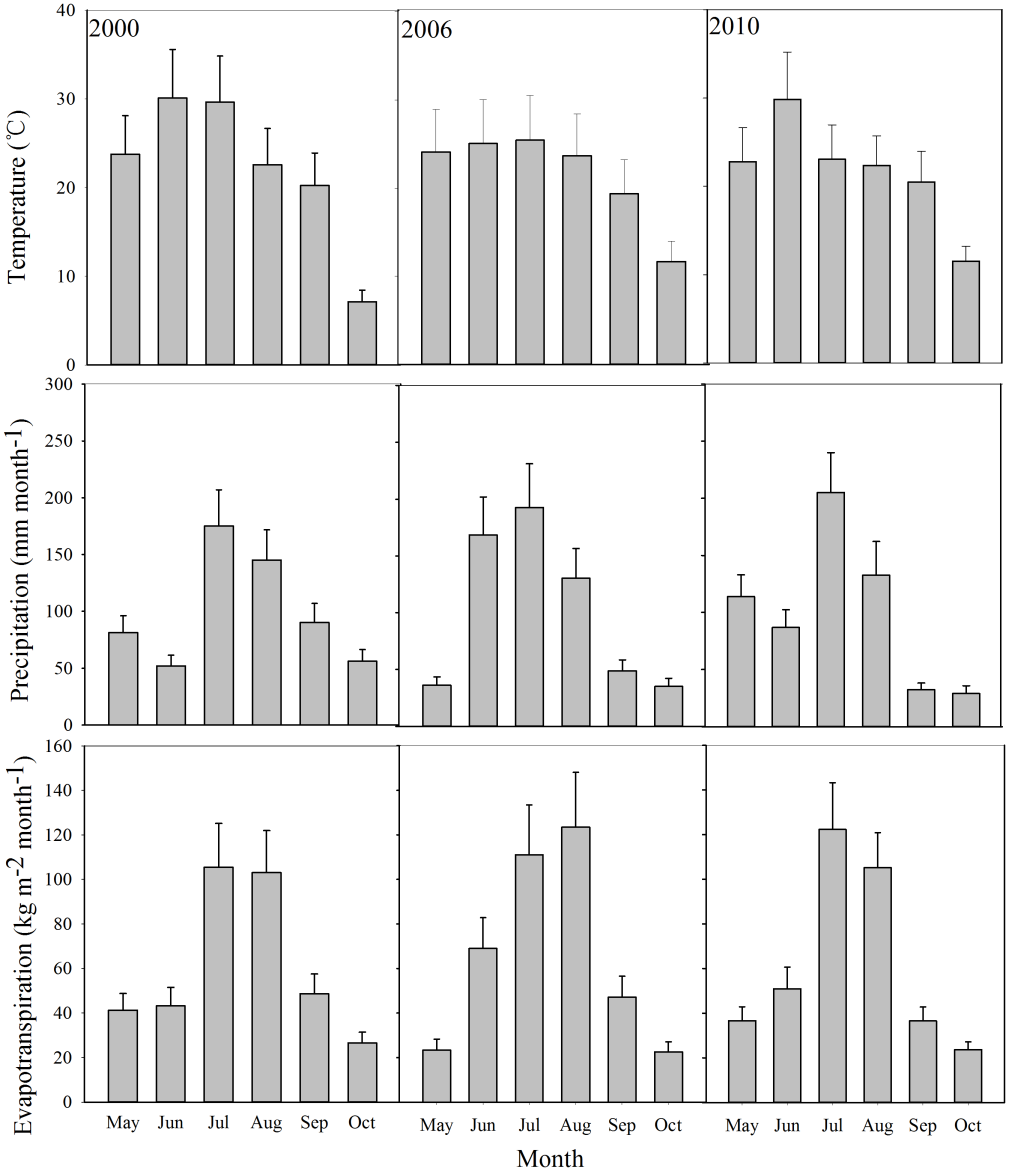
Seasonal changes of temperature, precipitation (P), and evapotranspiration (ET) from May to October.

**Fig 4:**
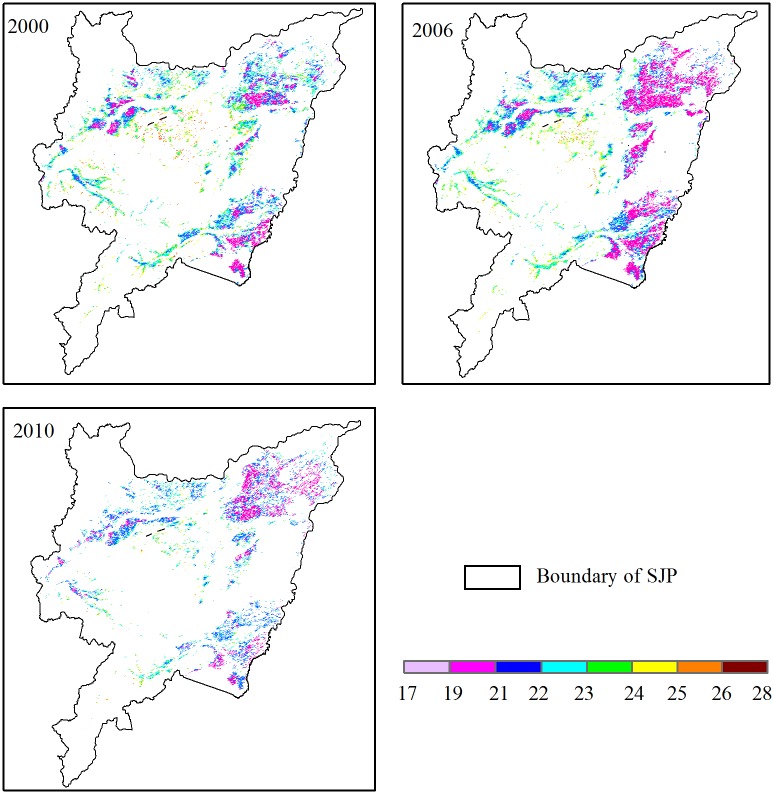
Spatial distribution of temperature (°C) in 2000, 2006, and 2010.

During the entire growing season from May to October, the average temperature was 22.08, 21.55, and 21.53°C in 2000, 2006, and 2010, respectively (Table 1). The low to high mean monthly temperature range was 7.13–30.1°C, 11.7–25.4°C, and 11.5–29.9°C during the entire growing season in 2000, 2006, and 2010, respectively. These figures show an obvious trend in the seasonal variation, with higher temperatures generally occurring in June and July (Fig 3). Temperature reached a maximum in June in 2000 and 2010, while the maximum temperature delayed for a month in 2006. The lowest temperature occurred in October during the rice growing seasons. The temperature distribution had obvious regional variation (Fig 4). The spatial range of the average temperature over the whole growing season was 18.3–27.6°C, 17.8–27.3°C, and 18.4–26.8°C in 2000, 2006, and 2010, respectively.

**Table 1:** Temperature and Ft from paddies of in the Sanjiang Plain (SJP).

Year	Temperature (°C)				Ft			
	Max	Min	Average	std	Max	Min	Average	std
**2000**	27.6	18.3	22.08	4.0	1.9	0.4	1.0	0.19
**2006**	27.3	17.8	21.55	4.3	2.1	0.4	1.0	0.20
**2010**	26.8	18.4	21.53	3.2	2.0	0.5	1.0	0.15

**Fig 5:**
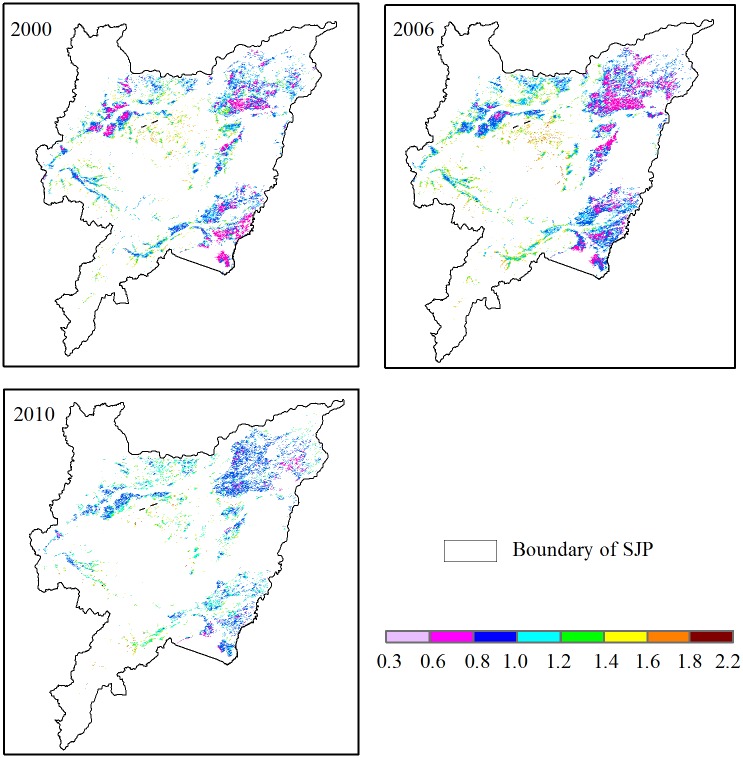
Spatial distribution of Ft in 2000, 2006, and 2010.

The factor Ft, which is an expression of the temperature, and displayed a decreasing trend from west to east. The Ft ranged from 0.41–1.93, 0.40–2.10, and 0.46–2.04 in the entire growing seasons of 2000, 2006, and 2010, respectively (Table 1). Relative higher Ft values (> 1.55) were located in a few pixels sparsely distributed in the downstream area of Ampang River, the Muling River Basin, and the Ken River Basin, accounting for 8.3, 6.9, and 4.4% of the whole SJP in 2000, 2006, and 2010, respectively (Fig 5). In contrast, lower values (< 1) for large patches of rice were concentrated in the east and the northwest, covering more than half of the whole rice growing area. In 2006, the maximum Ft was 2.1, which was higher than the maximum values in 2000 and 2010, which were 1.93 and 2.04, respectively. However, the minimum Ft in 2000 was much higher than in 2006 and 2010. The standard deviation of Ft was 0.19, 0.20, and 0.15 in 2000, 2006, and 2010, respectively.

**Fig 6:**
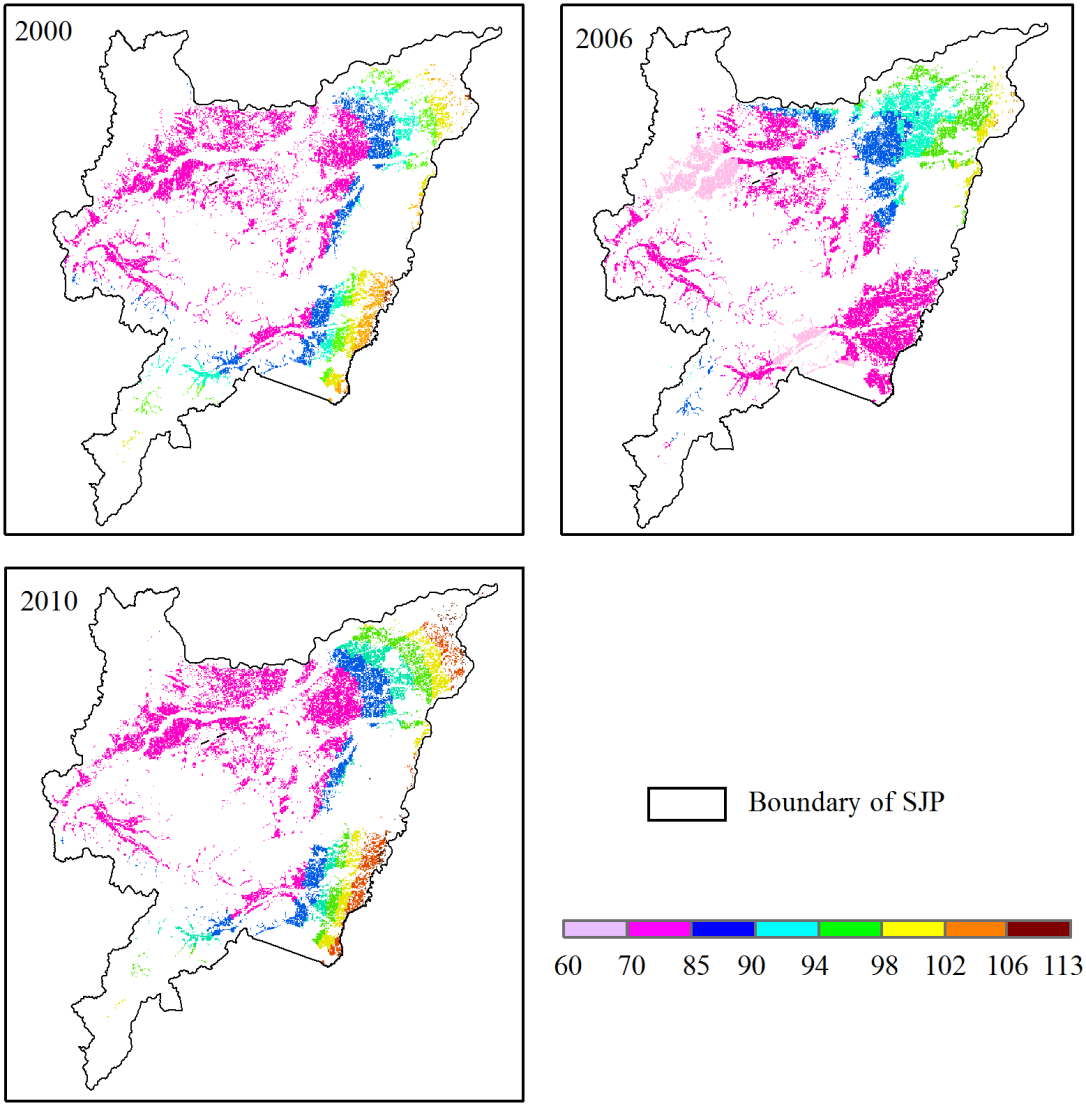
Spatial distribution of precipitation (P: mm month^-1^) in 2000, 2006 and 2010.

Mean precipitation was 85.1, 80.6, and 85.9 mm month^-1^ over the whole growing season in 2000, 2006, and 2010, respectively (Table 2). P had obvious regional and seasonal differences in the rice growing season, and presented a similar unimodal pattern of seasonal change to that of temperature (Figs 3 and 6). The low to high range of mean monthly P was 55.0–172.0, 35.2–187.2, and 28.0–198.1 mm month^-1^ during the whole growing season in 2000, 2006, and 2010, respectively (Fig 3). Large-intensity rainfall events were mainly concentrated in July and August, when P was close to or higher than 130 mm month^-1^ (Fig 3). P in the eastern areas was significantly higher than in the western areas in 2000, 2006, and 2010, respectively (Fig 6). The spatial range of the mean P was 61.5–106.8, 70.4–107.9, and 70.4–112.5 mm month^-1^ during the entire growing season in 2000, 2006, and 2010, respectively. Moreover, P showed a significant decreasing trend from the coast to inland.

**Table 2:** Evapotranspiration (ET), precipitation (P) and fw from paddies in the Sanjiang Plain (SJP).

Year	ET (mm month^-1^)				P (mm month^-1^)				fw			
	Max	Min	Average	std	Max	Min	Average	std	Max	Min	Average	std
**2000**	108.1	36.5	61.7	7.8	106.8	61.5	85.1	8.8	1.0	0.75	1.0	0.18
**2006**	100.6	39	66.5	7.1	107.9	70.4	80.6	9.8	1.0	0.70	1.0	0.20
**2010**	102.7	34.0	63.0	7.3	112.5	70.4	85.9	9.5	1.0	0.90	1.0	0.19

**Fig 7:**
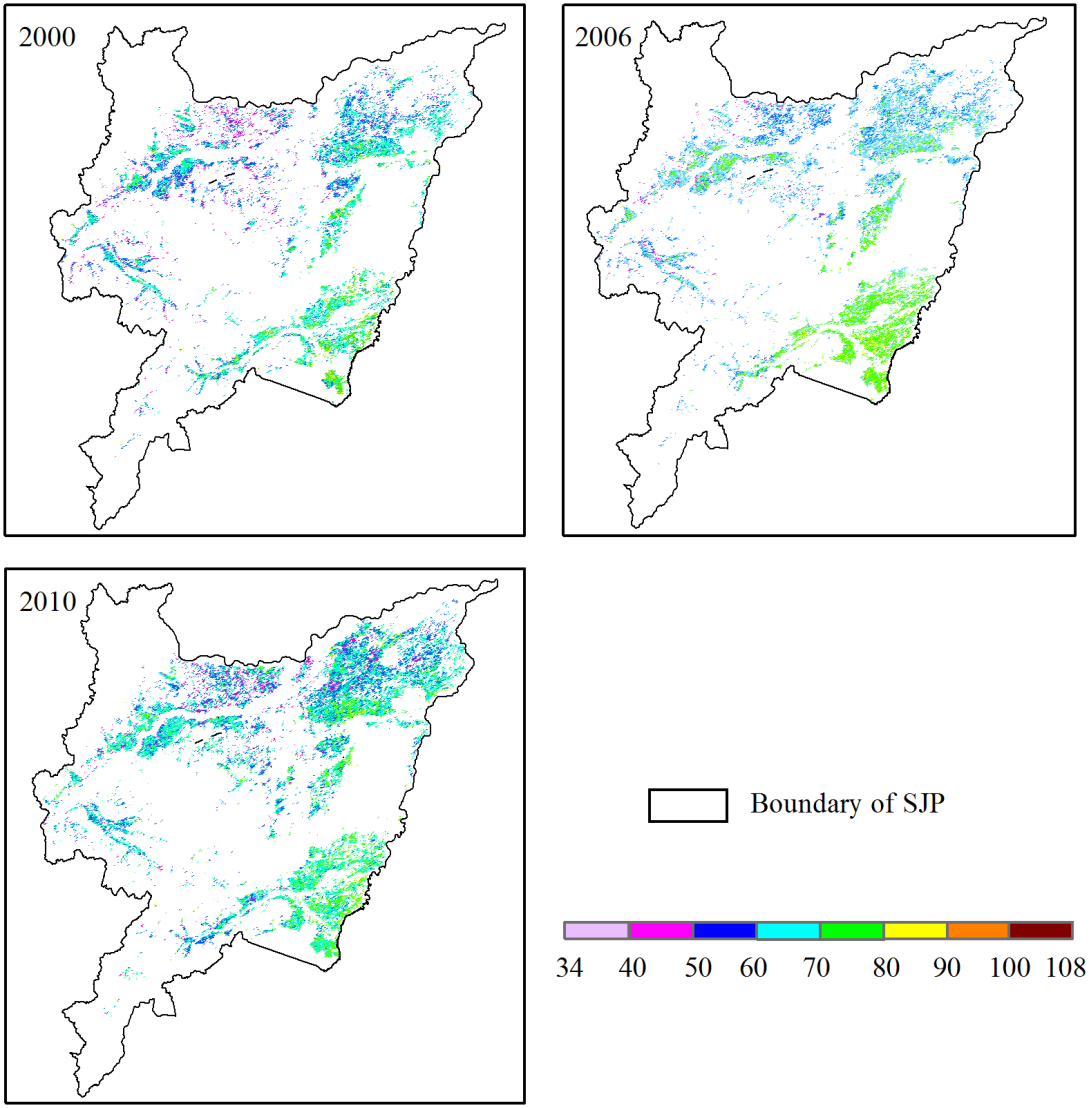
Spatial distribution of evapotranspiration (ET: mm month^-1^) in 2000, 2006, and 2010.

ET from rice fields had an obvious seasonal variation in accordance with the P in the SJP during the rice growing seasons of 2000, 2006, and 2010, respectively (Fig 3). The low to high range of mean monthly ET was 32.1–111.0, 28.9–129.5, and 14.5–160.0 mm month^-1^ during the whole growing season in 2000, 2006 and 2010, respectively (Fig 3). Changes in ET followed a similar trend to P and temperature, peaking in July and August. Due to the influence of solar radiation, temperature, wind speed, and other factors, the spatial distribution of ET was obvious in the SJP (Fig 7). High ET (> 62 mm month^-1^) occurred in the east in 2000. ET declined from east to west. In 2006, high ET was mainly observed in a broad area of the northeast. In 2010, ET was high in most parts of the SJP, and was higher than in 2000 and 2006.

### CH_4_ emissions in the SJP

**Fig 8:**
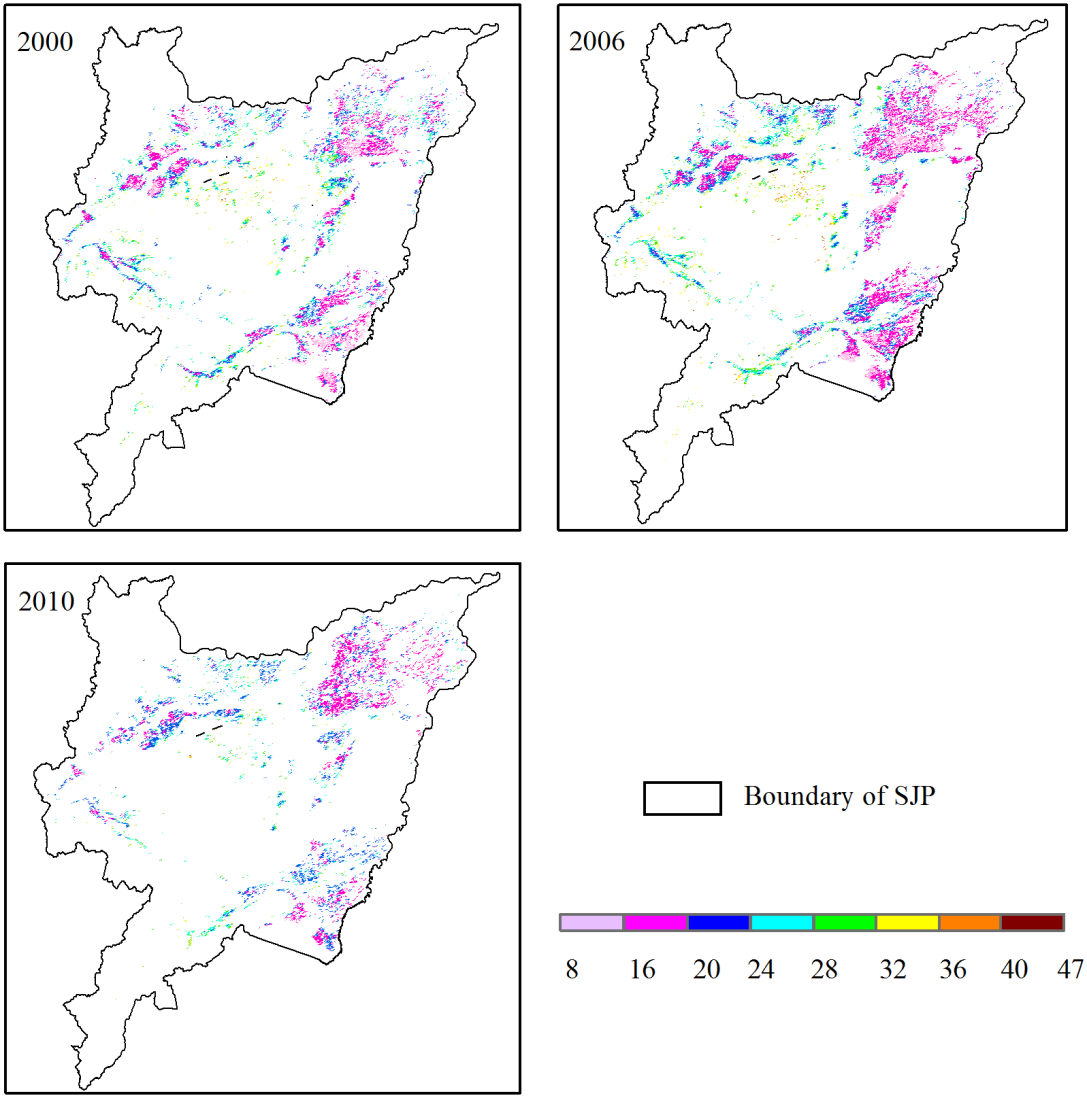
Spatial distribution of CH_4_ emissions (tons) from paddy rice at the 1 × 1 km grid cell scale in 2000, 2006, and 2010.

The statistical analysis showed that average CH_4_ emissions were 21.3 tons for every grid cell in 2010, which was higher than in 2000 (21.2 tons) and 2006 (21.0 tons) (Table 3). There were significant differences in the CH_4_ emissions among grid cells (Fig 8). Higher emissions (>32 tons) were found in the north-central area of the SJP, while lower emission rates were concentrated in the east and northwest areas. The distribution of CH_4_ emissions had clear regional differences (Fig 8). The spatial range of the CH_4_ emission was 9.9–41.7, 8.0–46.2, and 11.5–41.1 ton km^–2^ over the entire growing season of 2000, 2006, and 2010, respectively.

**Table 3:** Estimated results of CH_4_ emission from paddies of Sanjiang Plain (SJP).

Year	CH_4_ emission rates (ton km^–2^)			Total CH_4_ emission (Tg yr^-1^)
	Max	Min	Average	
**2000**	48.53	11.48	24.83	0.30
**2006**	53.84	9.32	24.63	0.36
**2010**	47.83	13.34	24.59	0.40

As a consequence of the local management practices for rice paddies in the SJP, the total CH_4_ emissions were estimated to be 0.30, 0.36, and 0.40 Tg yr^-1^ in the 2000, 2006, and 2010, respectively (Table 3). The CH_4_ emission rates ranged from 11.48 to 48.53 ton km^–2^ in 2000, with a mean of 24.83 ton km^–2^. The CH_4_ emission rates ranged from 9.32 to 53.84 ton km^–2^ in 2006, with a mean of 24.63 ton km^–2^. In 2010, the paddy rice emissions of CH_4_ were lower than in the other two years investigated at 24.59 ton km^–2^ (Table 3).

## Discussion

### Comparison of CH_4_ estimation with other studies

In this study, we used both relatively high resolution MODIS and TRMM data, such as LST, ET, and P, and field observed CH_4_ fluxes to estimate CH_4_ emissions during the growing season in the SJP. The spatial structure and magnitude of CH_4_ emissions were well-represented. We considered the effects of moisture and temperature on CH_4_ production in the process of CH_4_ estimation. Both the CH_4_ emissions and the environmental factors that influence them had an obvious spatial heterogenity in rice paddies. For rice paddy throughout the entire SJP, CH_4_ emissions were mainly concentrated around the 30 ton km^-2^ level, with this level accounting for 95, 93, and 98% of the entire planting area in 2000, 2006, and 2010. Higher (>32 ton km^-2^) and lower (<15 ton km^-2^) CH_4_ emissions accounted for less than 1% of the entire planting area. P and ET are important factors to measure the degree of soil water saturation [24]. However, the results show that for the artificial paddy field, the soil water content is saturated in both flooding and non-flooding periods. Therefore, the temperature is assumed the important role that contributed to the spatial variation of CH_4_ emissions from paddy fields. The temperature was mainly concentrated below 24°C, which accounted for 92, 95, and 98% of the entire planting area in 2000, 2006, and 2010. Higher (>25°C) and lower (<15°C) temperatures also accounted for less than 1% of the entire planting area.

The Intergovernmental Panel on Climate Change (IPCC) guidelines is considered to be a baseline assessment. The emissions calculated using the IPCC approach were 0.38, 0.45, and 0.50 Tg yr^-1^ in 2000, 2006, and 2010 [27]. Zhang *et al*. [11, 12] used the DNDC model to estimate methane emissions from rice paddies to be 0.50, 0.71, and 0.49 Tg yr^-1^ in SJP in 2000, 2006, and 2010, which were higher than our simulation results that were in the range of 0.35–0.46 Tg yr^-1^. The modeled CH_4_ emission rates varied over the range of 9.0–54.0 ton km^–2^ per year, which was consistent with the CH_4_ emission rates modeled by Zhang *et al*. in the SJP, which varied over the range of 3.0–60.0 ton km^–2^ per year [11].

### Uncertainties in CH_4_ estimation in SJP

We used a process-based methane emission model to evaluate the CH_4_ flux from rice fields in SJP. An advantage of this model is that it combined with a variety of environmental factors, and makes use of the remote sensing data. However, paddy field as an important anthropogenic source of atmospheric CH_4_, were different from natural wetlands. Variables considered in the model to evaluate the CH_4_ flux from rice fields may be environment factors and management practices [28]. Zhang et al (2011) pointed that the differences were indistinctive in rice cultivation practices making less variation in CH_4_ emissions from rice paddy in the SJP [14]. In this paper, we assumed the general (or average) management practices were identical in the entire study area. Some uncertainties existed in the modeling results since methane emissions consisted of three processes, i.e., generation, transmission, and emission. The accuracy of an estimation model is closely related to different factors (input parameters) affecting the various processes of CH_4_ emissions [29]. The greater the number of input parameters, the more detail may be provided regarding the mechanism of CH_4_ emission. In this study, there were some deficiency in the CH_4_ emission mechanism and parameter acquisition. We only considered the environmental factors, including temperature and moisture that affected CH_4_ production. It would be difficult to express the CH_4_ emission across a wide range of soil texture and other soil chemical properties [30–32]. Such a few of input parameters would result in some uncertainty in the estimated amount of CH_4_. In addition, we used the few observed CH_4_ flux that were available from previous studies in limited number of sites. The spatial scale limits of observed CH_4_ flux cannot adequately represent CH_4_ flux in the entire SJP, and would further contribute to the uncertainty of the CH_4_ estimation.

Temperature and ET estimated from MODIS data and P estimated from TRMM had a wide range of spatial heterogeneity. A higher spatial resolution of input parameters would express more detail in the spatial variation of the modeling results, and reduce uncertainties of the final estimation of CH_4_ emissions. In this study, we selected 1-km resolution remote sensing data, which was superior to other estimation methods based on regional or 10-km dimensions. However, there will still be spatial variations in temperature and ET be not detected. Remote sensing data as one of the input parameters in the model exists missing values in certain regions or periods. In this study, we found that the amount of missing data was limited, and we used the average value of the MODIS data to replace any missing data for the whole region. In addition, the spatial resolution of P data from the TRMM satellite is different other data. Scale mismatch between coarse resolutions of input data may result in substantial bias in estimation [33]. In addition, the satellite data products can be good inversion of meteorological data, but there is always a certain degree of deviation compared with the observed data. All of these treatments inevitably contributed to the uncertainty of the results. Further study will conduct to determine the impact of the quality of remote sensing data on regional CH_4_ emissions.

## Conclusions

Methane emissions from rice paddy fields in the SJP, China, were estimated based on remote sensing data. The results indicated that this method provided reliable estimates of the spatial distribution of CH_4_ emissions at the regional scale when compared with the IPCC approach and previous studies. Moisture and temperature were the main factors affecting the rice paddy CH_4_ emissions. The acquisition of high resolutions remote sensing data, such as ET, P and LST, would further improve the accuracy of CH_4_ emission estimations. The ET, P and LST products will be used to estimate CH_4_ emissions from rice paddies at different regional scales in future studies. The field observation of CH_4_ fluxes from paddy fields was an important factor that influenced the estimations. Therefore, field observation of CH_4_ fluxes that matched the remote sensing data will narrow the uncertainty of CH_4_ estimations.

## Supporting information

S1 File The data processing results. (RAR)

S1 Table Landsat TM imagery used for retrieving rice paddy. (PDF)

## References

[1] Houghton JT, Filho LGM, Callander BA. (1996) Climate change 1995: The science of climate change.

[2] Field CB, Barros VR, Mastrandrea MD, Mach KJ, Abdrabo MAK, Adger WN, et al (2015) Climate change 2014: Impacts, adaptation, and vulnerability. Part a: Global and sectoral aspects. Contribution of working group ii to the fifth assessment report of the intergovernmental panel on climate change. Guangdong Agricultural Sciences 285: 25987–25995.

[3] Smith P, Martino D, Cai ZC, Gwary D, Janzen H, Kumar P, et al (2008) Greenhouse gas mitigation in agriculture. Philosophical Transactions of the Royal Society of London 363: 789–813. 10.1098/rstb.2007.2184 17827109 PMC2610110

[4] Neue HU. (1993) Methane emission from rice fields. Bioscience 43: 466–474.

[5] Agnihotri S, Kulshreshtha K, Singh SN. (1999) Mitigation strategy to contain methane emission from rice-fields. Environmental Monitoring & Assessment 58: 95–104.

[6] Sass RL, Cicerone RJ. (2002) Photosynthate allocations in rice plants: Food production or atmospheric methane? Proceedings of the National Academy of Sciences of the United States of America 99: 11993–11995. 10.1073/pnas.202483599 12221297 PMC129384

[7] Ehhalt D, Prather M, Dentener F, Derwent R, Dlugokencky EJ, Holland E, et al. (2001) In Atmospheric chemistry and greenhouse gases, Related Information: Climate Change: Working Group I: the Scientific Basis: 228–248.

[8] Wang ZY, Xu YC, Li Z, Guo YX, Wassmann R, Neue HU, et al (2000) A four-year record of methane emissions from irrigated rice fields in the Beijing region of china. Nutrient Cycling in Agroecosystems 58: 55–63.

[9] Food and agriculture organisation of the united nations.

[10] Wang MX, Li J. (2002) CH_4_ emission and oxidation in chinese rice paddies. Nutrient Cycling in Agroecosystems 64: 43–55.

[11] Zhang Y, Su SL, Zhang F, Shi RH, Gao W. (2012) Characterizing spatiotemporal dynamics of methane emissions from rice paddies in northeast china from 1990 to 2010. Plos One 7: 108–108. 10.1371/journal.pone.0029156 PMC3250406 22235268

[12] Zhang Y, Wang YY, Su SL, Li CS. (2011) Quantifying methane emissions from rice paddies in northeast china by integrating remote sensing mapping with a biogeochemical model. Biogeosciences & Discussions 8: 1225–1235.

[13] Wang YY, Chen WW, Zhao ZC, Gu JX. (2008) Characteristics and estimation of CH_4_, N_2_O emissions from cold paddy field in the Sanjiang plain. Transactions of the Chinese Society of Agricultural Engineering 24: 170–176.

[14] Wang DX, Lu XG, Ding WX, Cai ZC, Wang YY. (2002) Comparison of methane emission from marsh and paddy field in Sanjiang plain. Scientia Geographica Sinica 22: 500–503.

[15] Chen WW, Wang YY, Zhao ZC, Feng C, Gu JX, Zheng XH. (2013) The effect of planting density on carbon dioxide, methane and nitrous oxide emissions from a cold paddy field in the Sanjiang Plain, northeast china. Agriculture Ecosystems & Environment 178: 64–70.

[16] Zou JW, Huang Y, Jiang JY, Zheng XH, Sass RL. (2005) A 3-year field measurement of methane and nitrous oxide emissions from rice paddies in china: Effects of water regime, crop residue, and fertilizer application. Global Biogeochemical Cycles 19: 153–174.

[17] Gutierrez J, Sang YK, Kim PJ. (2013) Effect of rice cultivar on CH4 emissions and productivity in korean paddy soil. Field Crops Research 146: 16–24.

[18] Khalil MAK, Rasmussen RA, Shearer MJ, Chen ZL, Yao H, Yang J. (1998) Emissions of methane, nitrous oxide, and other trace gases from rice fields in china. Journal of Geophysical Research Atmospheres 103: 25241–25250.

[19] Li CS, Frolking S, Frolking TA.(1992) A model of nitrous oxide evolution from soil driven by rainfall events: 2. Model applications. Journal of Geophysical Research Atmospheres 97: 9777–9783.

[20] Giltrap DL, Li C, Saggar S. (2010) DNDC: A process-based model of greenhouse gas fluxes from agricultural soils. Agriculture Ecosystems & Environment 136: 292–300.

[21] Lobell DB, Lesch SM, Corwin DL, Ulmer MG, Anderson KA, Potts DJ, et al (2010) Regional-scale assessment of soil salinity in the red river valley using multi-year MODIS EVI and NDVI. Journal of Environmental Quality 39: 35–41. 10.2134/jeq2009.0140 20048292

[22] Shamsi SRF, Zare S, Abtahi SA. (2012) Soil salinity characteristics using moderate resolution imaging spectroradiometer (MODIS) images and statistical analysis. Archives of Agronomy & Soil Science 59: 1–19.

[23] Agarwal R, Garg JK. (2007) Methane emission modelling using MODIS thermal and optical data: A case study on Gujarat. Journal of the Indian Society of Remote Sensing 35:323–331.

[24] Akumu CE, Pathirana S, Baban S, Bucher D. (2010) Modeling methane emission from wetlands in north-eastern new south wales, australia using landsat etm+. Remote Sensing 2: 855–860.

[25] Xu H, Cai ZC, Tsuruta H. (2003) Soil moisture between rice-growing seasons affects methane emission, production, and oxidation. Soil Science Society of America Journal 67: 1147–1157.

[26] Xie BH, Zheng XH, Zhou ZX, Gu JX, Zhu B, Chen X, et al (2010) Effects of nitrogen fertilizer on CH4 emission from rice fields: Multi-site field observations. Plant & Soil 326: 393–401.

[27] Paustian K, Ravindranath NH,Amstel AR. (2006) 2006 IPCC guidelines for national greenhouse gas inventories 2: 48–56

[28] Yan XY, Kazuyuki Y, Hiroko A, Hajime A. (2005) Statistical analysis of the major variables controlling methane emission from rice fields. Global Change Biology 11: 1131–1141.

[29] Zhu Q, Liu J, Peng C, Chen H, Fang X, et al (2014) Modelling methane emissions from natural wetlands by development and application of the TRIPLEX-GHG model. Geoscientific Model Development 7: 981–999.

[30] Aulakh MS, Wassmann R, Rennegberg H. (2001) Methane emissions from rice field—quantification, mechanisms, role of management, and mitigation options.Advances in Agronomy 70: 193–260.

[31] Mer Le, Roger P (2001) Production, oxidation, emission and consumption of methane by soils: a review. European Journal of Soil Biology 37: 25–50.

[32] Yan X, Ohara T, Akimoto H. (2003) Development of regionspecific emission factors and estimation of methane emission from rice fields in the East, Southeast and South Asian countries. Global Change Biology 9: 237–254.

[33] Feng F, Li XL, Yao YJ, Liang SL, Chen JQ and Zhao X, et al (2016) An Empirical Orthogonal Function-Based Algorithm for Estimating Terrestrial Latent Heat Flux from Eddy Covariance, Meteorological and Satellite Observations. Plos one 11:e0160150 10.1371/journal.pone.0160150 27472383 PMC4966955

