# Supplementary material for: Satellite data based estimation of methane emissions from rice paddies in the Sanjiang Plain in northeast China
Source: PLoS One. 2017 Jun 6;12(6):e0176765. doi: 10.1371/journal.pone.0176765 (PMC5460792; doi:10.1371/journal.pone.0176765)
Supplement: S1 Table — (PDF) [file pone.0176765.s002.pdf]

S1 Table Landsat TM imagery used for retrieving rice paddy.

Path/Row No.	Acquisition date		
	**2000**	**2006**	**2010**
113/26			19/09/2010
113/27	05/09/2002		19/09/2010
113/28			19/09/2010
113/29			19/09/2010
114/26		30/08/2006	
114/27	11/08/2002	30/08/2006	06/06/2010
114/28	25/09/2001	30/08/2006	06/06/2010
114/29	25/09/2001	30/08/2006	
115/27	12/08/2000	22/09/2006	14/09/2009, 17/09/2010
115/28	12/08/2000	22/09/2006	14/09/2009
115/29	31/08/2001	22/09/2006	14/09/2009
116/27	07/09/2001	31/08/2006	08/09/2010
116/28	07/09/2001	31/08/2006	08/00/2010
